# Disrupted Neurogenesis in Germ-Free Mice: Effects of Age and Sex

**DOI:** 10.3389/fcell.2020.00407

**Published:** 2020-05-29

**Authors:** Gavin A. Scott, Dylan J. Terstege, Alex P. Vu, Sampson Law, Alexandria Evans, Jonathan R. Epp

**Affiliations:** Cumming School of Medicine, Hotchkiss Brain Institute, Department of Cell Biology and Anatomy, University of Calgary, Calgary, AB, Canada

**Keywords:** neurogenesis, hippocampus, microbiome, germ-free, proliferation

## Abstract

The gut microbiome has profound effects on development and function of the nervous system. Recent evidence indicates that disruption of the gut microbiome leads to altered hippocampal neurogenesis. Here, we examined whether the effects of gut microbiome disruption on neurogenesis are age-dependent, given that both neurogenesis and the microbiome show age-related changes. Additionally, we examined memory induced functional connectivity of hippocampal networks. Control and germ-free mice at three different ages (4, 8, and 12 weeks) were trained in contextual fear-conditioning, then subsequently tested the following day. Hippocampal neurogenesis, quantified via BrdU and doublecortin, exhibited age-dependent changes relative to controls, with the established age-dependent decrease in neurogenesis being delayed in germ-free mice. Moreover, we found sex-dependent effects of germ-free status on neurogenesis, with 4 week old male germ-free mice having decreased neurogenesis and 8 week old female germ-free mice having increased neurogenesis. To assess systems-level consequences of disrupted neurogenesis, we assessed functional connectivity of hippocampal networks by inducing c-Fos expression with contextual memory retrieval and applying a previously described network analysis. Our results indicate impaired connectivity of the dentate gyrus in germ-free mice in a pattern highly correlated with adult neurogenesis. In control but not germ-free mice, functional connectivity became more refined with age, indicating that age dependent network refinement is disrupted in germ-free mice. Overall, the results show that disruption of the gut microbiome affects hippocampal neurogenesis in an age- and sex-dependent manner and that these changes are also related to changes in the dentate gyrus functional network.

## Introduction

Emerging evidence indicates that the gut microbiome plays a substantial role in cognition due to direct and indirect communication with the brain via the gut-brain axis (Dinan and Cryan, 2017; Sarkar et al., 2018). In humans, gut microbiome diversity is correlated with cognitive performance (Arnoriaga-Rodríguez and Fernández-Real, 2019) and supplementation with probiotics has been shown to improve cognition (Allen et al., 2016). Additionally, numerous rodent studies now report impaired cognition when the microbiome is disrupted (Gareau et al., 2011; Wang et al., 2015; Fröhlich et al., 2016; Möhle et al., 2016) and improved cognition resulting from probiotic supplementation (Savignac et al., 2015; Wang et al., 2015; Gronier et al., 2018).

Notably, evidence indicates that the gut microbiome is also linked with depression and anxiety (Foster and McVey Neufeld, 2013). In humans, supplementation with probiotics has been shown to alleviate low mood (Benton et al., 2007). Disruptions of the gut microbiome via infection or inflammation have also been shown to increase anxiety-like behavior (Lyte et al., 2006; Goehler et al., 2008). Fecal transplants from subjects with a depressed or anxious phenotype to normal subjects can also transfer these mood impairments (Bercik et al., 2011; Kelly et al., 2016). Germ-free mice, which are devoid of gut microbiota, show a reduction in basal anxiety behaviors (Neufeld et al., 2011), but germ-free status leads to heightened HPA responses to acute stress in both rats (Crumeyrolle-Arias et al., 2014) and mice (Clarke et al., 2013).

Recent studies have established that adult hippocampal neurogenesis and behavior can change with perturbations in the gut microbiome (Ogbonnaya et al., 2015; Möhle et al., 2016). Neurogenesis has been heavily implicated in multiple cognitive processes relating to learning, memory, and cognitive flexibility (Winocur et al., 2006; Sahay et al., 2011; Epp et al., 2016). Furthermore, it is also implicated in anxiety- and depression-related behavior (Duman, 2004). Reduced neurogenesis is observed in rodents subjected to chronic stress (Lucassen et al., 2015). Additionally, antidepressant drugs increase neurogenesis, which appears to be necessary for mediating the improvement in depression-related behaviors in mice (Santarelli et al., 2003). Thus, the relationship between the gut microbiome and anxiety/depression may be mediated in part by changes in neurogenesis.

Importantly, both neurogenesis and the gut microbiome undergo age-dependent changes. Rates of neurogenesis decrease sharply with age (Kuhn et al., 1996; Amrein et al., 2004). The composition and the diversity of gut microbiota increase during postnatal development (Eckburg et al., 2005; Claesson et al., 2011). Hence, the relationship between neurogenesis, the gut microbiome, and anxiety- and depression-like behavior may be further modulated by the age of the animal.

In the present study, we compared rates of neurogenesis in the dentate gyrus between germ-free and control mice at different ages to determine whether the relationship between neurogenesis and gut microbiota changed with age. We also trained animals in contextual fear conditioning at these ages in order to assess age-modulated differences between control and germ-free mice in the expression of fear memory, which is related to anxiety and depression. Furthermore, we applied a graph theoretical approach to examine task-specific networks of neuronal activation during the expression of this fear memory to determine how changes in neurogenesis and fear memory expression might coincide with altered functional connectivity between the DG and other brain areas. This approach allows us to determine the impact of altered neurogenesis on brain connectivity. Relatively little previous research has examined the link between the microbiome and neurogenesis (Ogbonnaya et al., 2015; Möhle et al., 2016) and no previous study, to our knowledge, has examined age as an independent variable in this context.

## Methods

### Animals

A total of 45 control C57BL/6J and 45 germ-free C57BL/6J mice were purchased from Charles River (Wilmington, MA, United States) and the International Microbiome Facility (IMC) (University of Calgary, Canada). To produce the germ-free line, C57BL/6J mice were re-derived to germ-free status via two-cell embryo transfer. Axenic mice were bred and maintained long-term in flexible-film isolators at the IMC. Germ-free status was routinely monitored by culture-dependent and -independent methods and all germ-free colonies were independently confirmed to be pathogen-free. Germ-free status was maintained until the first behavioral experiments. Specifically, behavioral procedures began on the same day that germ-free animals were brought into the laboratory from the suppliers. Male and female mice from three age groups (4 weeks old, 8 weeks old, and 12 weeks old) were housed in groups of 5 and provided food and water *ad libitum*. All mice were housed under a 12-h light/12-h dark cycle. Mice were used in accordance with protocols approved by the University of Calgary, Health Sciences Animal Care Committee, following guidelines of the Canadian Council for Animal Care.

### Contextual Fear Conditioning

Mice were trained in contextual fear conditioning. Training was conducted in a sound-attenuated chamber (Ugo Basile, Gemonio, Italy) with a grated floor from which shocks (0.5 mA; 2 s) were delivered. Behavior was monitored via an overhead camera and automated tracking software (ANY-Maze, Stoelting, Wood Dale, IL, United States). During the training phase, mice were allowed to explore the chamber for 2 min before a series of 3 shocks were delivered with a 1 min interval between each shock. The addition of ∼500 μL of bleach into the test chamber provided an additional olfactory cue. Mice were returned to the chamber 24 h after training for a 5-min retention test in which no shocks were delivered. The chamber was cleaned using 70% ethanol and allowed to dry after each animal.

### Perfusions and Histology

Ninety minutes after retention testing in contextual fear conditioning, animals were perfused with 0.1 M phosphate buffered saline (PBS) followed by 4% formaldehyde. Brains were extracted and postfixed in 4% formaldehyde for 24 h. Fixed brains were then stored at 4°C in 30% W/V sucrose until they were no longer buoyant. Serial sections were collected on a cryostat (Leica Biosystems, Concord, ON, Canada) at a thickness of 40 μm and stored in 10 series at −20°C in antifreeze solution.

### Immunohistochemistry

#### Doublecortin Labeling

Tissue sections were washed 3 times in 0.1 M PBS before being placed in a primary antibody solution containing 1:200 rabbit anti-DCX (4604S, Cell Signaling Technology, Danvers, MA, United States), 0.03% Triton-X, and 3% normal donkey serum and incubated for 48 h. Tissue sections then underwent 3 10-min PBS washes before being placed in a secondary antibody solution containing 1:500 donkey anti-goat Alexa Fluor 488 antibody (CLAS10-1116, Cedarlane Labs, Burlington, ON, Canada) and incubated for 24 h. The subsequent day, tissue sections were incubated in a 1:2000 dilution of 4,6-diamidino-2-phenylindole (DAPI) for at least 10 min before being mounted to slides and coverslipped using PVA-DABCO mounting medium.

#### BrdU Labeling

Two hours before perfusion, mice were weighed and given a single intraperitoneal injection of 200 mg kg^–1^ BrdU (B-5002, Sigma Aldrich, Oakville, ON, Canada) dissolved in 20 mg/ml sterile saline. Following perfusion and tissue sectioning, brain sections were washed 3 times in 0.1 M PBS, then incubated in a 45°C oven in 2N HCl for 30 min to denature DNA. The HCl was then neutralized by rinsing sections using 1 M sodium borate buffer (pH 8.5) for 10 min followed by 3 × 10 min washes in 0.1 M PBS. BrdU was labeled by incubating sections for 48 h at 4°C with 1:250 mouse monoclonal anti-BrdU primary antibody (Bu20a, BioLegend, San Diego, CA, United States) in blocking solution (3% normal donkey serum, 0.03% Triton-X in 0.1 M PBS). Sections were rinsed 3 times in 0.1 M PBS then incubated at 4°C with a donkey anti-mouse secondary antibody conjugated to Alexa Fluor 488 (715-545-150, Jackson ImmunoResearch Laboratories Inc., West Grove, PA, United States) diluted 1:500 in 0.1 M PBS. Tissue was then transferred to a 1:3000 solution of propidium iodide for 10 min. Tissue was rinsed with PBS before mounting in PVA-DABCO.

#### C-Fos Labeling

Tissue sections were washed 3 times in 0.1 M PBS before being transferred to a primary antibody solution containing 1:2000 rabbit anti-cfos antibody (226 003, Synaptic Systems, Göttingen, Germany), 3% normal donkey serum, and 0.03% Triton-X and were incubated at room temperature for 48 h. Tissue sections were then washed 3 times in PBS and transferred to a secondary antibody solution containing 1:500 donkey anti-rabbit Alexa Fluor 488 (111-545-003, Cedarlane Labs, Burlington, ON, Canada) secondary antibody and incubated for 24 h. Sections were then transferred to 1:2000 DAPI and incubated for 15 min before being mounted to slides and coverslipped with PVA-DABCO mounting medium.

### Quantification of Neurogenesis and Pyknosis

Neurogenesis was quantified by counting the number of doublecortin and BrdU positive cells in the subgranular zone (SGZ) and granule cell layer of the DG. Labeled cells were identified using a 60× oil immersion objective on an Olympus FLUOVIEW FV3000 confocal microscope (Richmond Hill, ON, Canada) by an experimenter blind to the subject age, sex, and germ-free status. Approximately 7–10 sections per brain were sampled and exhaustive counts of every positive cell were obtained for each section. For DCX, brains were quantified unilaterally. The number of positive cells was standardized to the area of the DG, which in the case of DCX quantification was captured using a 2× objective lens with 2× zoom and, in the case of BrdU quantification, was captured with a 10× objective. The area of the DG was quantified via manual tracing in *ImageJ* software (United States NIH). Pyknotic cells were imaged by staining a separate series of tissue sections with cresyl violet and were counted exhaustively in the same manner. We operationally defined pyknotic cells as those exhibiting darker staining and condensed chromatin in the nucleus (Falconer and Galea, 2003; Pawluski et al., 2010). In order to avoid counting cell caps, we also counted only the cells that were surrounded by translucent cytoplasm and were not situated at the extreme upper or lower focal planes of the section. The area of the DG for cresyl violet-stained sections was quantified by capturing images on an Olympus VS120-L100-W slide scanner (Richmond Hill, ON, Canada) and the DG in these images was manually traced in *ImageJ*.

### Functional Connectivity of Hippocampal Networks

Analysis of functional connectivity was performed via an automated process that we developed, which builds upon analyses of correlated regional cFos expression density (Wheeler et al., 2013). In brief, tissue sections stained for cFos expression were imaged using an Olympus VS120-L100-W slide scanner (Richmond Hill, ON, Canada). Regional cFos expression density was measured using a semi-automated image processing pipeline. Fluorescent cFos labels were segmented using the machine learning-based pixel and object classification program *Ilastik* (Berg et al., 2019). Images were then registered to a selection of regions from the Allen Mouse Brain Atlas (Region list and abbreviations are provided in Supplementary Table 1) using a custom and user input-driven *ImageJ* plug-in. The regional c-Fos densities were then correlated within each group to construct pairwise correlation matrices. To generate a binary adjacency matrix, correlations were filtered by an alpha value of 0.95 and only statistically significant correlations with a Pearson’s *r* of at least 0.8 were considered. In such a matrix, all comparisons in which the filter criteria were met are denoted with a one while all other comparisons are denoted with a zero. Binary adjacency matrices can then be analyzed as network graphs by plotting all regions being analyzed and connecting all pairs of regions which were marked with a one in the adjacency matrix. A graph theoretical approach guided by the use of the Brain Connectivity Toolbox (Rubinov and Sporns, 2010) was used to analyze measures of network connectivity and generate graphs of each network in an automated manner.

Among these measures, node degree and global network density were highlighted. In the case of our neuroanatomical networks, each region is defined as a node and correlated activity between a pair of regions is represented by a vertex between nodes (Bullmore et al., 2009). Node degree signifies the connectedness of a node and is calculated by counting the number of vertices connected to that node. Network density extends upon this and is expressed as a proportion of the total number of possible vertices in a graph with an equivalent number of nodes (Achard and Bullmore, 2007).

### Statistical Analysis

All statistical tests for neurogenesis, pyknosis, and behavior in the fear conditioning test were performed using Statistica (version 13 TIBCO software). To analyze the differences between the groups, a two-way ANOVA followed by a Newman-Keuls multiple comparisons *post hoc* test was utilized. To detect statistically significant differences between the groups a *p*-value of 0.05 was set as the threshold for significance. The analysis of functional networks was performed as per the described procedure above. Brains were excluded from tissue analyses if the quality of the tissue was insufficient (e.g., poor perfusion or damaged sections) or lacked adequate expression of BrdU. In all cases, exclusion occured blind to condition and prior to quantification to avoid bias.

## Results

### Germ-Free Mice Show Altered Adult Neurogenesis

**FIGURE 1:**
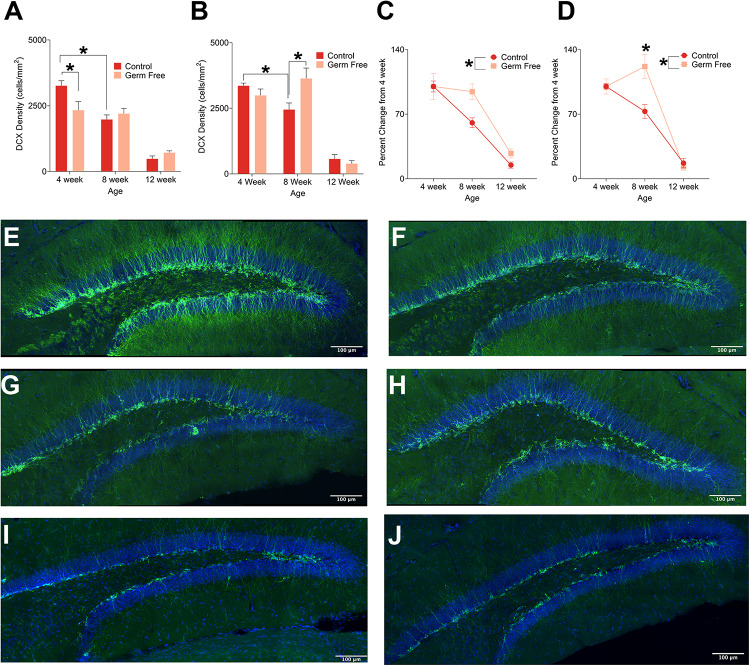
**(A)** Mean (± SEM) DCX-positive cells in the DG of male germ-free and control mice. Control mice show a clear age-dependent decrease in neurogenesis, but this pattern is altered in germ-free mice. At 4 weeks old, male germ-free mice had reduced neurogenesis relative to controls and exhibited no reduction in neurogenesis between 4 and 8 weeks of age. **(B)** Mean (± SEM) DCX-positive cells in the DG of female germ-free and control mice. Similarly to males, female control mice showed a clear age-related decrease in neurogenesis with this effect being absent in female germ-free mice. In contrast to male germ-free mice, female germ-free mice showed no difference relative to controls at 4 weeks old, but had significantly elevated neurogenesis at 8 weeks old. **(C)** Mean (± SEM) neurogenesis in males depicted as percent-change from the baseline (4 week old) number of DCX-positive DG cells. Neurogenesis remains abnormally elevated in germ-free mice as they age relative to controls **(D)**. Mean (± SEM) neurogenesis in females depicted as percent-change from the baseline (4 week old) number of DCX-positive DG cells. As with male germ-free mice, neurogenesis in female germ-free mice remains abnormally elevated relative to controls particularly at 8 weeks of age. **(E–J)** Representative photomicrographs of DCX-positive cells (green) and DAPI (blue) in the DG of 4 week old controls **(E)** and germ-free mice **(F)**, 8 week old controls **(G)** and germ-free mice **(H)**, and 12 week old controls **(I)** and germ-free mice **(J)**. Control male 4 week *n* = 11, 8 week *n* = 11, 12 week *n* = 8. Control female 4 week *n* = 5, 8 week, *n* = 5, 12 week *n* = 4. Germ-free male 4 week *n* = 11, 8 week *n* = 11, 12 week *n* = 6. Germ-free female 4 week *n* = 5, 8 week, *n* = 5, 12 week *n* = 4. *p < 0.05.

To examine how the gut microbiome might impact adult hippocampal neurogenesis we quantified DCX, a marker of immature neurons, within the DG of the hippocampus in germ-free and control mice. Representative photomicrographs of DCX-positive cells are shown in Figures 1E–J. Because adult neurogenesis is not a static process we performed this analysis at three different ages, 4, 8 or 12 weeks of age. As expected, our results demonstrated a statistically significant decline in doublecortin-labeled cells in control mice with increasing age. This effect was evident in both male (Figure 1A; *F*(2,51) = 44.97, *p* < 0.0001) and female mice (Figure 1B; *F*(2,22) = 68.84, *p* < 0.0001). However, in germ-free mice, the same relationship between age and doublecortin was not observed and the result was sexually dimorphic. In males, there was a significant decrease in doublecortin at 4 weeks in germ-free mice compared to control mice (*p* = 0.006) but no difference at 8 or 12 weeks (*p*’s > 0.498). In female mice we observed a significant increase in doublecortin in 8 week old germ-free mice compared to control mice (*p* = 0.014) and no significant differences at 4 or 12 weeks of age (*p*’s > 0.307). To compare the rate of change of doublecortin-labeling across male and female germ-free and control mice, we examined the percent change in labeling from the respective 4 week old mice. In doing so we observed that in both male and female germ-free mice, the decline in neurogenesis that occurs between 4 and 8 week old control mice was absent in germ-free mice [Male (Figure 1C): significant main effect of group *F*(1,51) = 4.43, *p* = 0.04, Female (Figure 1D): Significant group × age interaction (*F*(2,22) = 6.79, *p* < 0.0050)]. In the case of females, there was even a small but significant increase in doublecortin labeling between 4 and 8 weeks (*p* = 0.028).

### Germ-Free Mice Show Altered Cell Proliferation

**FIGURE 2:**
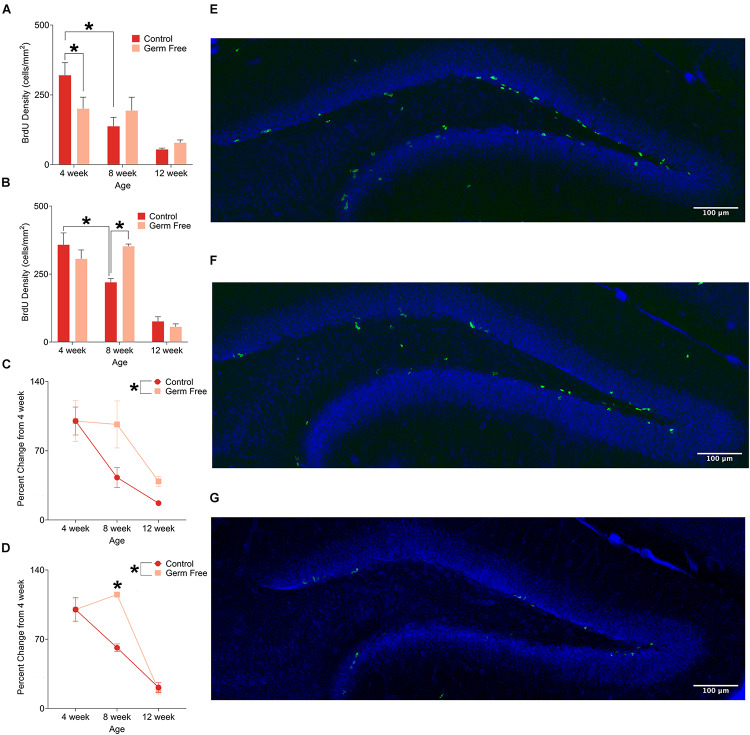
**(A)** Mean (± SEM) BrdU-positive cells in the DG of male germ-free and control mice. Control mice show a clear pattern of age-related decline in cell proliferation, whereas germ-free mice show no such reduction between 4 and 8 weeks of age. Germ-free mice also show reduced cell proliferation relative to controls at 4 weeks of age. **(B)** Mean (± SEM) BrdU-positive cells in the DG of female germ-free and control mice. As with males, female control mice show an age-related decline in cell proliferation with this effect being absent in germ-free mice between 4 and 8 weeks of age. Moreover, female germ-free mice have increased cell proliferation relative to controls at 8 weeks of age. **(C)** Mean (± SEM) cell proliferation in males depicted as percent-change from the baseline (4 week old) number of BrdU-positive DG cells. Cell proliferation remains abnormally elevated in germ-free mice as they age relative to controls **(D)**. Mean (± SEM) cell proliferation in females depicted as percent-change from the baseline (4 week old) number of BrdU-positive DG cells. As with male germ-free mice, cell proliferation in female germ-free mice remains abnormally elevated relative to controls particularly at 8 weeks of age. **(E–G)** Representative photomicrographs of BrdU-positive cells (green) and propidium iodide (blue) in the DG of 4 week old **(E)**, 8 week old **(F)**, and 12 week old **(G)** control mice illustrating the age-related decrease in cell proliferation. Control male 4 week *n* = 10, 8 week *n* = 7, 12 week *n* = 8. Control female 4 week *n* = 4, 8 week, *n* = 5, 12 week *n* = 4. Germ-free male 4 week *n* = 5, 8 week *n* = 7, 12 week *n* = 8. Germ-free female 4 week *n* = 4, 8 week, *n* = 3, 12 week *n* = 3. *p < 0.05.

In addition to the number of immature neurons, we also measured cell proliferation by quantifying BrdU in the dentate gyrus. Representative photomicrographs of BrdU-positive cells are shown in Figures 2E–G. Again, as expected we identified a significant decrease in proliferation with age in control mice for both males (Figure 2A; significant interaction of Age^∗^Group: *F*(2,39) = 3.53, *p* = 0.038) and females (Figure 2B; significant interaction of Age^∗^Group: *F*(2,15) = 5.09, *p* = 0.021). However, in germ-free mice the age dependent decrease in neurogenesis was disrupted, following the same pattern as observed for DCX labeling. That is, a decrease in proliferation at 4 weeks in male mice (*p* = 0.018) and an increase in proliferation at 8 weeks in female mice (*p* = 0.017). As a function of percent change from 4 weeks of age, male (Figure 2C) and female (Figure 2D) germ-free mice showed a flat or slight increase in proliferation between 4 and 8 weeks of age, respectively, compared to control mice that show a decrease in both sexes over this time [Male: significant main effect of group (*F*(1,39) = 4.45, *p* = 0.041), Female: Significant group × age interaction (*F*(2,15) = 5.96, *p* < 0.012)].

### Germ-Free Mice Have Increased Cell Death in the Dentate Gyrus at 4 Weeks of Age

**FIGURE 3:**
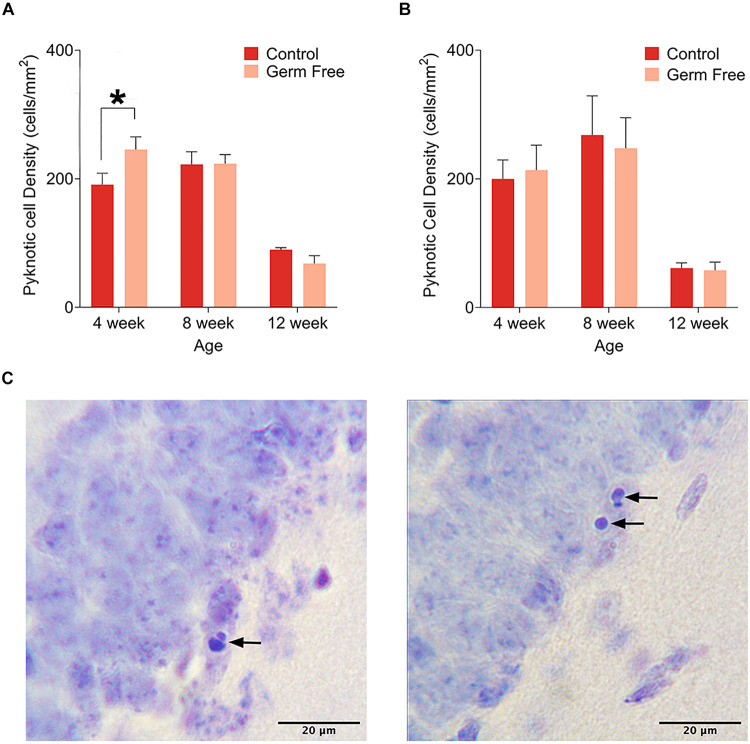
**(A)** Mean (± SEM) pyknotic cells in the DG of male germ-free and control mice. The number of pyknotic cells is highest in 4 week old animals and lowest in 12 week old animals. Additionally, germ-free mice show an increased rate of cell death at 4 weeks of age. **(B)** Mean (± SEM) pyknotic cells in the DG of female germ-free and control mice. As is the case in male mice, the rate of cell death is highest in 4 week old animals and lowest in 12 week old animals. There is also a slight trend toward elevated cell death at 8 weeks of age. In contrast to male mice, female germ-free mice showed no change in the rate of cell death relative to controls. Control male 4 week *n* = 10, 8 week *n* = 10, 12 week *n* = 9. Control female 4 week *n* = 4, 8 week, *n* = 4, 12 week *n* = 4. Germ-free male 4 week *n* = 9, 8 week *n* = 9, 12 week *n* = 10. Germ-free female 4 week *n* = 4, 8 week, *n* = 4, 12 week *n* = 4. **(C)** Representative photomicrographs of DG cells with pyknotic morphology. *p < 0.05.

As a measure of cell death, we quantified the number of pyknotic cells in the dentate gyrus. Representative images of cresyl violet-stained pyknotic cells are shown in Figure 3A. We observed a pattern of cell death across ages in both male (Figure 3B) and female (Figure 3C) control mice that replicated the previously described pattern of reduced cell death across age (Sun et al., 2004) and was slightly reminiscent of the previously described inverted U pattern of cell death across age (Ben Abdallah et al., 2010), although the rate of pyknosis was very similar between 4 and 8 weeks with only slight increases in males and females that did not reach statistical significance. Mainly, the results show a sharp reduction in the rate of pyknosis at 12 weeks compared to 4 or 8 weeks (*p*’s ≤ 0.000165). In male germ-free mice, there was a significant group by age interaction (*F*(2,51) = 4.07, *p* = 0.023) in the density of pyknotic cells. *Post hoc* tests showed a significant difference between control and germ-free mice at 4 weeks of age (*p* = 0.025). There was no significant main effect of group (*p* = 0.91) or group by age interaction (*p* = 0.90) in female mice but there was a significant main effect of age (*F*(2,18) = 14.87, *p* = 0.00015). 12 week old mice had significantly fewer pyknotic cells than 4 week (*p* = 0.0012) and 8 week old mice (*p* = 0.00028).

### Germ-Free Mice Show Reduced Functional Connectivity of the Dentate Gyrus

**FIGURE 4:**
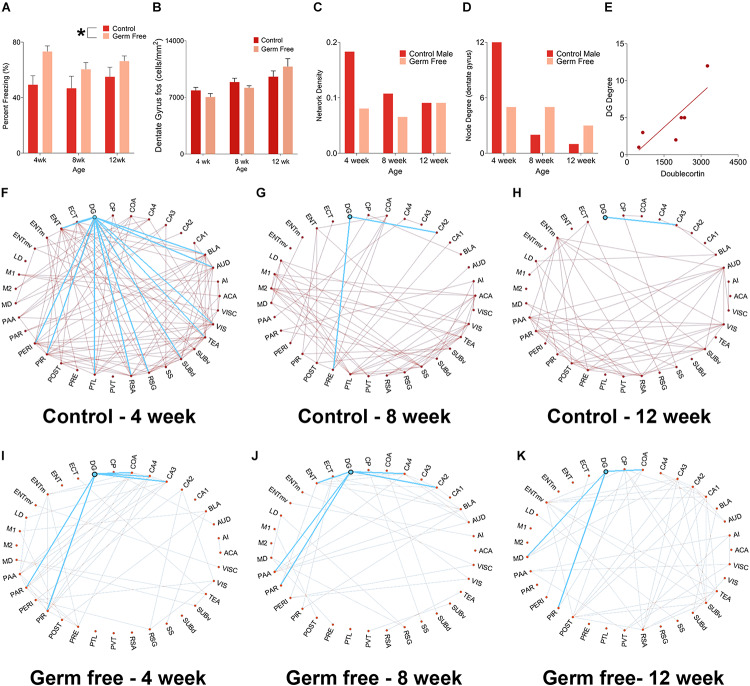
**(A)** Mean (± SEM) percent freezing in contextual fear conditioning in male mice. Germ-free mice froze significantly more than control mice, indicating enhanced expression of fear memory. The effect was most pronounced in 4-week-old mice. **(B)** Mean (± SEM) c-fos-positive cells per mm^2^ in the DG of germ-free and control mice. There was no difference in absolute c-fos expression between groups. However, c-fos expression increased significantly with age. **(C)** Network density, expressed as the ratio of number of connections:total possible connections, in control and germ-free mice. Control mice exhibited an age-related decrease in network density, whereas germ-free mice had an initially reduced network density which did not decrease with age. **(D)** Node degree of the DG in germ-free and control mice. Controls show a large decrease in node degree as they age, indicating a refinement of the DG network as it exhibits a progressive reduction in the number of regions it has correlated activity with. The DG of germ-free mice has an initial reduction in node degree relative to controls and, in contrast to controls, does not undergo a reduction in its node degree as a function of age, indicating that the DG of germ-free mice maintains correlated activity with a greater number of regions across age than in controls. **(E)** A scatterplot showing the correlation between DG node degree and DCX expression. The node degree of the DG is positively correlated with DCX expression, suggesting the possibility that increased neurogenesis may drive an increase in the number of regions with which the DG has correlated activity. **(F–H)** Network graphs for control mice at 4 weeks old **(F)**, 8 weeks old **(G)**, and 12 weeks old **(H)** with the functional connectivity of the DG shown in blue. Control mice show a decrease in both network density and DG node degree across ages. **(I–K)**. Network graphs for germ-free mice at 4 weeks old **(F)**, 8 weeks old **(G)**, and 12 weeks old **(H)** with the functional connectivity of the DG highlighted. Germ-free mice show no change across ages in network density or DG node degree. Control male 4 week *n* = 6, 8 week *n* = 5, 12 week *n* = 7. Germ-free male 4 week *n* = 5, 8 week *n* = 7, 12 week *n* = 5. *p < 0.05.

We next sought to determine the impact that altered rates of hippocampal neurogenesis have on correlated activity with other brain regions. To do so, we used a c-fos-based approach to determine functional connectivity. This technique, which has been used previously, is based on detection of correlated activity between regions within a group of mice. In order to induce c-fos activity we perfused mice 90 min following fear memory recall. For this analysis, we used male mice only because we observed highly variable behavior in female mice due to typical periodic bouts of darting behavior which interfere with the functional connectivity interpretations. Interestingly, germ-free mice spent significantly more time freezing during the contextual memory test in all age groups (Figure 4A, main effect of treatment, *F*(1,55) = 10.49, *p* = 0.0002). The difference appeared most pronounced in 4-week-old mice but, there was no significant effect of age (*F*(2,55) = 0.98, *p* = 0.38) or Age by Group interaction (*F*(2,55) = 0.59, *p* = 0.56). There was no difference in the absolute number of c-fos-positive cells in the DG (cells/mm^2^) between controls and germ-free mice although c-fos expression was greater in older mice than younger mice (Figure 4B and Supplementary Figure 1; significant main effect of Age: *F*(2,28) = 11.50, *p* = 0.0002). Based on pairwise correlations of c-fos activity (Figure 4C) across mice we next examined alterations in functional connectivity. Control mice exhibited a decrease in network density with increasing age (i.e., total number of functional connections in the network). The decrease in network density across age was non-linear, with the greatest change occurring between 4 and 8 weeks of age (Figure 4C). This indicated a refinement in the network in older mice. In germ-free mice, on the other hand, the network density remained stable across ages suggesting an impairment in maturation of the hippocampal networks (Figure 4D). In addition, we looked specifically at the connectivity of the dentate gyrus and observed, in control mice, an age-dependent decrease in the number of regions exhibiting significantly correlated activity with the dentate gyrus (Figures 4F–H). However, in germ-free mice, there was a reduced number of functionally connected regions in the youngest age group and this level of connectivity was relatively constant with age (Figures 4I–K). Because the network properties are determined per group rather than per mouse we correlated the node degree of the DG for each group (i.e., number of functionally connected regions) with the group mean doublecortin densities to determine the relationship between neurogenesis and DG functional connectivity. We found a significant correlation between DG node degree and the number of doublecortin labeled neurons (Figure 4E, r(4) = 0.83, *p* = 0.043).

## Discussion

In the present experiment, we examined whether alteration of the gut microbiome exerts age-dependent changes on neurogenesis, HPC-dependent memory, and the functional connectivity of hippocampal networks. We found that the established (Kuhn et al., 1996; Amrein et al., 2004) pattern of age-related changes in neurogenesis was altered in germ-free mice, with the classic sharp decline in postnatal neurogenesis being delayed in germ-free mice relative to controls. These results are partially consistent with previous research showing that disruptions of the gut microbiome can alter neurogenesis (Ogbonnaya et al., 2015; Möhle et al., 2016). We extend these previous findings by showing that microbiome-related alteration in neurogenesis is age-dependent, with differences in neurogenesis between germ-free and controls appearing to normalize as animals age. The effects of disrupted gut microbiota on neurogenesis may therefore be most critical in younger animals.

Our results partially replicate an aspect of a previous report examining neurogenesis in germ-free mice (Ogbonnaya et al., 2015) found that neurogenesis in germ-free mice was elevated at 10 weeks of age. We found elevation of both BrdU- and DCX-positive cells specifically in female germ-free mice at 8 weeks of age. Conversely, we found decreased neurogenesis in male germ-free mice at 4 weeks of age. Overall, our findings indicate that neurogenesis is not uniformly elevated in germ-free mice and that this effect is both age- and sex-dependent. However, when we analyzed rates of neurogenesis as a percent change from baseline, germ-free status in both sexes leads to the same basic pattern of a delayed age-related reduction in neurogenesis as a result of germ-free status.

The causes of the complex pattern of results across age and sex cannot be determined from the present experiment, but the pattern of neurogenesis is similar to that of Gobinath et al. (2017). These authors treated nursing rat dams with either corticosterone (CORT) or vehicle and found that neurogenesis in the dorsal HPC of the offspring declined more slowly in both males and females and that neurogenesis was initially lower in males compared to the offspring of vehicle-treated dams. Previous research has shown that serum levels of CORT are elevated in germ-free rats (Crumeyrolle-Arias et al., 2014). Thus, the present results could potentially be explained in part by differences in serum CORT concentration which shows similar age- and sex-dependent effects on neurogenesis (Gobinath et al., 2017).

The absence of gut microbiota causes a range of effects in addition to increasing CORT such as alterations in serotonin biosynthesis (Yano et al., 2015) and hippocampal serotonergic signaling (Clarke et al., 2013) which has been shown to play a role in regulating neurogenesis (Alenina and Klempin, 2015). Additionally, disruption of the gut microbiome has been shown to impair neurogenesis through a mechanism involving Ly6Chi monocytes (Möhle et al., 2016). The gut microbiome’s role in the maturation of microglia (Thion et al., 2018), another cell type with influence on hippocampal neurogenesis (Stefani et al., 2018), could act as an additional pathway between the gut and the brain. Hence, there are multiple mechanisms that could be driving the effects we presently observe of germ-free status on neurogenesis.

To determine whether altered neurogenesis was accompanied by altered rates of cell death, we also quantified the number of pyknotic cells in the DG. Across both sexes, the rate of DG cell death was higher in younger animals than in older animals, consistent with previous findings (Sun et al., 2004; Ben Abdallah et al., 2010). Interestingly, male germ-free mice had increased cell death at 4 weeks of age whereas this effect was absent in females. Although the mechanisms underlying this sex difference are unclear, this effect could potentially be related to the decrease in neurogenesis in our 4 week old male germ-free mice, a decrease that was not present in female germ-free mice at this age. However, the pattern of cell death was largely similar between germ-free and control mice, indicating that germ-free status had much less influence on cell death than it did on neurogenesis.

We also examined the behavior of germ-free mice in contextual fear conditioning and found that germ-free mice had an increased freezing response during retention testing, indicating an enhancement of fear memory expression. The difference was greatest at 4 weeks of age with smaller increases in the freezing of 8-week-old and 12-week-old mice. Previous research in rodents has shown that, generally, learning and memory is impaired following disruption of the gut microbiome (Gareau et al., 2011; Wang et al., 2015; Fröhlich et al., 2016; Möhle et al., 2016). The results may be explained by an increase in anxiety-like behavior. Although some previous research has found that germ-free mice exhibit decreased basal anxiety (Neufeld et al., 2011), germ-free status causes heightened HPA responses to induced stress (Clarke et al., 2013; Crumeyrolle-Arias et al., 2014). Our present behavioral findings may thus be explained by an increased neuroendocrine response to footshock stress.

We also examined functional connectivity of hippocampal networks in male mice. As control animals aged, they exhibited a decrease in the density of network connections. In contrast, germ-free mice exhibited relatively stable network density at all ages examined, although network density in germ-free was lower than controls at the younger ages. When we examined the functional connectivity of the dentate gyrus specifically, we identified an age related decrease in connectivity in control mice but this trend was altered in germ-free mice. In germ-free mice, the connectivity was initially reduced in 4 week old mice but remained relatively stable between 4 and 8 weeks of age. Activity in the DG is very sparse with most cells being unresponsive to any spatial context (Jung and McNaughton, 1993; Alme et al., 2010). This limited size of the “functional” pool of DG cells may lead to a reduced opportunity for correlated activity with other brain regions and a more sparse functional network. It has been proposed that neurogenesis replenishes the functional pool of DG cells (Lisman, 2011) and, indeed, newly born DG neurons are more active than older DG neurons in response to environmental enrichment (for example Tashiro et al., 2007). Thus, the lack of a decrease in the degree of DG functional connectivity in germ-free mice may be explained by the delay in the age-related decrease of neurogenesis. In fact, we found a strong correlation between doublecortin labeling and dentate gyrus node degree which accounts for 68% of the variability in dentate connectivity. These results indicate that under control conditions, the functional network involving the DG becomes more sparse over the course of development consistent with increasing refinement and path efficiency (Bullmore et al., 2009; Rubinov and Sporns, 2010). In germ-free mice, and very possibly as a result of disrupted neurogenesis, this “refinement” of functional networks is impaired and this may form part of the mechanism of impaired cognition in germ-free animals. Importantly, the present findings are correlational, and further experiments involving ablation or enhancement of neurogenesis would be required to establish that these functional connectivity changes in germ-free mice are causally related to neurogenesis.

A secondary but noteworthy finding from our functional connectivity analysis was the lack of functional connectivity between the DG and the entorhinal cortex. Given the dense anatomical connections between these two regions and that functional connectivity is often strongly predicted by anatomical connectivity (Goñi et al., 2014), this finding is rather surprising. However, anatomical connectivity does not always predict functional connectivity (Honey et al., 2009). We are also not the first to observe little or no functional connectivity between the DG and entorhinal cortex after a 24 h retention interval in contextual fear conditioning, whereas a 4 week retention interval does evoke functional connectivity between the DG and entorhinal cortex (Wheeler et al., 2013; Vetere et al., 2017). Thus, different task parameters may result in stronger functional connectivity between the two regions.

The pattern of functional connectivity that we observed may also have been influenced by the fact that animals were tested post-BrdU injection and injection stress may have affected the pattern of neuronal activation. We used BrdU as a method for measuring cell proliferation in order to align with the methods of Ogbonnaya et al. (2015). However, all mice received BrdU injections and therefore effects of BrdU administration should be consistent across groups.

We examined the age- and sex-dependent effects of germ-free status on hippocampal neurogenesis, and functional connectivity of hippocampal networks. We show that germ-free status delays the normal age-related decline in neurogenesis and that this effect was also sex-dependent. The results show that there is an important age component to the effects of the gut microbiome on hippocampal neurogenesis. Specifically, alterations in neurogenesis as a result of microbiome dysfunction may be most apparent in younger animals. Moreover, this effect is sexually dimorphic, with male germ-free mice initially having reduced rates of neurogenesis at 4 weeks and female germ-free mice having elevated neurogenesis at 8 weeks. We also show that the development and maturation of the DG functional network is disrupted with germ-free status, an effect that seems reflected in the lack of age-dependent changes seen in the neurogenesis of germ-free animals and represents a major, systems-level alteration in functional connectivity as a consequence of germ-free status. Given the strong correlation between neurogenesis and node degree, these results indicate that disruption of the gut microbiome may be driven to a major extent by disrupted neurogenesis. Thus, disrupted neurogenesis may be a major mechanism through which gut dysbiosis causes cognitive impairments particularly early in neurodevelopment.

## Data Availability Statement

The raw data supporting the conclusions of this article will be made available by the authors, without undue reservation, to any qualified researcher.

## Ethics Statement

Experiments were conducted in accordance with the Canadian Council on Animal Care guidelines were approved by the University of Calgary Health Sciences Animal Care Committee.

## Author Contributions

AV, GS, and SL conducted the behavioral experiments. AV, GS, DT, SL, and AE performed the histology and image analysis. DT performed the network analysis. AV, DT, and JE performed the data analysis. AV, GS, DT, and JE conceived the experiments and wrote the manuscript.

## Conflict of Interest

The authors declare that the research was conducted in the absence of any commercial or financial relationships that could be construed as a potential conflict of interest.

## References

[B1] Achard S. Bullmore E. (2007). Efficiency and cost of economical brain functional networks. *PLoS Comput. Biol.* 3:e17. 10.1371/journal.pcbi.0030017 17274684 PMC1794324

[B2] Alenina N. Klempin F. (2015). The role of serotonin in adult hippocampal neurogenesis. *Behav. Brain Res.* 277 49–57. 10.1016/j.bbr.2014.07.038 25125239

[B3] Allen A. P. Hutch W. Borre Y. E. Kennedy P. J. Temko A. Boylan G. (2016). Bifidobacterium longum 1714 as a translational psychobiotic: modulation of stress, electrophysiology and neurocognition in healthy volunteers. *Transl. Psychiatry* 6:e939. 10.1038/tp.2016.191 27801892 PMC5314114

[B4] Alme C. B. Buzzetti R. A. Marrone D. F. Leutgeb J. K. Chawla M. K. Schaner M. J. (2010). Hippocampal granule cells opt for early retirement. *Hippocampus* 20 1109–1123. 10.1002/hipo.20810 20872737

[B5] Amrein I. Slomianka L. Poletaeva I. I. Bologova N. V. Lipp H. P. (2004). Marked species and age-dependent differences in cell proliferation and neurogenesis in the hippocampus of wild-living rodents. *Hippocampus* 14 1000–1010. 10.1002/hipo.20018 15390172

[B6] Arnoriaga-Rodríguez M. Fernández-Real J. M. (2019). Microbiota impacts on chronic inflammation and metabolic syndrome - related cognitive dysfunction. *Rev. Endocr. Metab. Disord.* 20 473–480. 10.1007/s11154-019-09537-5 31884557

[B7] Ben Abdallah N. M.-B. Slomianka L. Vyssotski A. L. Lipp H.-P. (2010). Early age-related changes in adult hippocampal neurogenesis in C57 mice. *Neurobiol. Aging* 31 151–161. 10.1016/j.neurobiolaging.2008.03.002 18455269

[B8] Benton D. Williams C. Brown A. (2007). Impact of consuming a milk drink containing a probiotic on mood and cognition. *Eur. J. Clin. Nutr.* 61 355–361. 10.1038/sj.ejcn.1602546 17151594

[B9] Bercik P. Denou E. Collins J. Jackson W. Lu J. Jury J. (2011). The intestinal microbiota affect central levels of brain-derived neurotropic factor and behavior in mice. *Gastroenterology* 141 599–609. 21683077 10.1053/j.gastro.2011.04.052

[B10] Berg S. Kutra D. Kroeger T. Straehle C. N. Kausler B. X. Haubold C. (2019). ilastik: interactive machine learning for (bio)image analysis. *Nat. Methods* 16 1226–1232. 10.1038/s41592-019-0582-9 31570887

[B11] Bullmore E. Sporns O. Bullmore E. Sporns O. Sporns O. (2009). Complex brain networks: graph theoretical analysis of structural and functional systems. *Nat. Rev. Neurosci.* 10 186–198. 10.1038/nrn2575 19190637

[B12] Claesson M. J. Cusack S. O’Sullivan O. Greene-Diniz R. De Weerd H. Flannery E. (2011). Composition, variability, and temporal stability of the intestinal microbiota of the elderly. *Proc. Natl. Acad. Sci. U.S.A.* 108 4586–4591. 20571116 10.1073/pnas.1000097107 PMC3063589

[B13] Clarke G. Grenham S. Scully P. Fitzgerald P. Moloney R. D. Shanahan F. (2013). The microbiome-gut-brain axis during early life regulates the hippocampal serotonergic system in a sex-dependent manner. *Mol. Psychiatry* 18 666–673. 10.1038/mp.2012.77 22688187

[B14] Crumeyrolle-Arias M. Jaglin M. Bruneau A. Vancassel S. Cardona A. Daugé V. (2014). Absence of the gut microbiota enhances anxiety-like behavior and neuroendocrine response to acute stress in rats. *Psychoneuroendocrinology* 42 207–217. 10.1016/j.psyneuen.2014.01.014 24636517

[B15] Dinan T. G. Cryan J. F. (2017). The microbiome-gut-brain axis in health and disease. *Gastroenterol. Clin. North Am.* 46 77–89. 28164854 10.1016/j.gtc.2016.09.007

[B16] Duman R. S. (2004). Depression: a case of neuronal life and death? *Biol. Psychiatry* 56 140–145. 10.1016/j.biopsych.2004.02.033 15271581

[B17] Eckburg P. B. Bik E. M. Bernstein C. N. Purdom E. Dethlefsen L. Sargent M. (2005). Diversity of the human intestinal microbial flora. *Science* 308 1635–1638. 10.1126/science.1110591 15831718 PMC1395357

[B18] Epp J. R. Silva Mera R. Kohler S. Josselyn S. A. Frankland P. W. (2016). Neurogenesis-mediated forgetting minimizes proactive interference. *Nat. Commun.* 7 5–12. 10.1038/ncomms10838 PMC4773435 26917323

[B19] Falconer E. M. Galea L. A. M. (2003). Sex differences in cell proliferation, cell death and defensive behavior following acute predator odor stress in adult rats. *Brain Res.* 975 22–36. 10.1016/s0006-8993(03)02542-3 12763590

[B20] Foster J. A. McVey Neufeld K. A. (2013). Gut-brain axis: how the microbiome influences anxiety and depression. *Trends Neurosci.* 36 305–312. 10.1016/j.tins.2013.01.005 23384445

[B21] Fröhlich E. E. Farzi A. Mayerhofer R. Reichmann F. Jačan A. Wagner B. (2016). Cognitive impairment by antibiotic-induced gut dysbiosis: analysis of gut microbiota-brain communication. *Brain Behav. Immun.* 56 140–155. 10.1016/j.bbi.2016.02.020 26923630 PMC5014122

[B22] Gareau M. G. Wine E. Rodrigues D. M. Cho J. H. Whary M. T. Philpott D. J. (2011). Bacterial infection causes stress-induced memory dysfunction in mice. *Gut* 60 307–317. 10.1136/gut.2009.202515 20966022

[B23] Gobinath A. R. Workman J. L. Chow C. Lieblich S. E. Galea L. A. M. (2017). Sex-dependent effects of maternal corticosterone and SSRI treatment on hippocampal neurogenesis across development. *Biol. Sex Differ.* 8 1–13. 28580124 10.1186/s13293-017-0142-x PMC5454586

[B24] Goehler L. E. Park S. M. Opitz N. Lyte M. Gaykema R. P. A. (2008). Campylobacter jejuni infection increases anxiety-like behavior in the holeboard: possible anatomical substrates for viscerosensory modulation of exploratory behavior. *Brain Behav. Immun.* 22 354–366. 10.1016/j.bbi.2007.08.009 17920243 PMC2259293

[B25] Goñi J. van den Heuvel M. P. Avena-Koenigsberger A. Velez de Mendizabal N. Betzel R. F. Griffa A. (2014). Resting-brain functional connectivity predicted by analytic measures of network communication. *Proc. Natl. Acad. Sci. U.S.A.* 111 833–838. 10.1073/pnas.1315529111 24379387 PMC3896172

[B26] Gronier B. Savignac H. M. Di Miceli M. Idriss S. M. Tzortzis G. Anthony D. (2018). Increased cortical neuronal responses to NMDA and improved attentional set-shifting performance in rats following prebiotic (B-GOS^®^) ingestion. *Eur. Neuropsychopharmacol.* 28 211–224. 10.1016/j.euroneuro.2017.11.001 29174530 PMC5857269

[B27] Honey C. J. Sporns O. Cammoun L. Gigandet X. Thiran J. P. Meuli R. (2009). Predicting human resting-state functional connectivity from structural connectivity. *Proc. Natl. Acad. Sci. U.S.A.* 106 2035–2040. 10.1073/pnas.0811168106 19188601 PMC2634800

[B28] Jung M. W. McNaughton B. L. (1993). Spatial selectivity of unit activity in the hippocampal granular layer. *Hippocampus* 3 165–182. 10.1002/hipo.450030209 8353604

[B29] Kelly J. R. Borre Y. O’ Brien C. Patterson E. El Aidy S. Deane J. (2016). Transferring the blues: depression-associated gut microbiota induces neurobehavioural changes in the rat. *J. Psychiatr. Res.* 82 109–118. 10.1016/j.jpsychires.2016.07.019 27491067

[B30] Kuhn H.-G. Dickinson-Anson H. Gage F. H. (1996). Neurogenesis in the dentate gyrus of the adult rat: age-related decrease of neuronal progenitor proliferation. *J. Neurosci.* 16 2027–2033. 10.1523/jneurosci.16-06-02027.1996 8604047 PMC6578509

[B31] Lisman J. (2011). Formation of the non-functional and functional pools of granule cells in the dentate gyrus: role of neurogenesis, LTP and LTD. *J. Physiol.* 589 1905–1909. 10.1113/jphysiol.2010.201137 21098002 PMC3090593

[B32] Lucassen P. J. Oomen C. A. Naninck E. F. G. Fitzsimons C. P. van Dam A.-M. Czeh B. (2015). Regulation of adult neurogenesis and plasticity by (early) stress, glucocorticoids, and inflammation. *Cold Spring Harb. Perspect. Biol.* 7:a021303. 10.1101/cshperspect.a021303 26330520 PMC4563706

[B33] Lyte M. Li W. Opitz N. Gaykema R. P. A. Goehler L. E. (2006). Induction of anxiety-like behavior in mice during the initial stages of infection with the agent of murine colonic hyperplasia Citrobacter rodentium. *Physiol. Behav.* 89 350–357. 10.1016/j.physbeh.2006.06.019 16887154

[B34] Möhle L. Mattei D. Heimesaat M. M. Bereswill S. Fischer A. Alutis M. (2016). Ly6Chi monocytes provide a link between antibiotic-induced changes in gut microbiota and adult hippocampal neurogenesis. *Cell Rep.* 15 1945–1956. 10.1016/j.celrep.2016.04.074 27210745

[B35] Neufeld K. M. Kang N. Bienenstock J. Foster J. A. (2011). Reduced anxiety-like behavior and central neurochemical change in germ-free mice. *Neurogastroenterol. Motil.* 23 255–264. 10.1111/j.1365-2982.2010.01620.x 21054680

[B36] Ogbonnaya E. S. Clarke G. Shanahan F. Dinan T. G. Cryan J. F. O’Leary O. F. (2015). Adult hippocampal neurogenesis is regulated by the microbiome. *Biol. Psychiatry* 78 e7–e9. 10.1016/j.biopsych.2014.12.023 25700599

[B37] Pawluski J. L. Barakauskas V. E. Galea L. A. M. (2010). Pregnancy decreases oestrogen receptor alpha expression and pyknosis, but not cell proliferation or survival, in the hippocampus. *J. Neuroendocrinol.* 22 248–257. 10.1111/j.1365-2826.2010.01960.x 20136685

[B38] Rubinov M. Sporns O. (2010). Complex network measures of brain connectivity: uses and interpretations. *Neuroimage* 52 1059–1069. 10.1016/j.neuroimage.2009.10.003 19819337

[B39] Sahay A. Scobie K. N. Hill A. S. O’Carroll C. M. Kheirbek M. A. Burghardt N. S. (2011). Increasing adult hippocampal neurogenesis is sufficient to improve pattern separation. *Nature* 472 466–470. 10.1038/nature09817 21460835 PMC3084370

[B40] Santarelli L. Saxe M. Gross C. Surget A. Battaglia F. Dulawa S. (2003). Requirement of hippocampal neurogenesis for the behavioral effects of antidepressants. *Science* 301 805–809. 10.1126/science.1083328 12907793

[B41] Sarkar A. Harty S. Lehto S. M. Moeller A. H. Dinan T. G. Dunbar R. I. M. (2018). The microbiome in psychology and cognitive neuroscience. *Trends Cogn. Sci.* 22 611–636. 29907531 10.1016/j.tics.2018.04.006

[B42] Savignac H. M. Tramullas M. Kiely B. Dinan T. G. Cryan J. F. (2015). Bifidobacteria modulate cognitive processes in an anxious mouse strain. *Behav. Brain Res.* 287 59–72. 10.1016/j.bbr.2015.02.044 25794930

[B43] Stefani J. Tschesnokowa O. Parrilla M. Robaye B. Boeynaems J.-M. Acker-Palmer A. (2018). Disruption of the MICROGLIAL ADP receptor p2y13 enhances adult hippocampal neurogenesis. *Front. Cell. Neurosci.* 12:134. 10.3389/fncel.2018.00134 29867367 PMC5966569

[B44] Sun W. Winseck A. Vinsant S. Park O. H. Kim H. Oppenheim R. W. (2004). Programmed cell death of adult-generated hippocampal neurons is mediated by the proapoptotic gene bax. *J. Neurosci.* 24 11205–11213. 10.1523/jneurosci.1436-04.2004 15590937 PMC6730275

[B45] Tashiro A. Makino H. Gage F. H. (2007). Experience-specific functional modification of the dentate gyrus through adult neurogenesis: a critical period during an immature stage. *J. Neurosci.* 27 3252–3259. 10.1523/jneurosci.4941-06.2007 17376985 PMC6672473

[B46] Thion M. S. Low D. Silvin A. Chen J. Grisel P. Schulte-Schrepping J. (2018). Microbiome influences prenatal and adult microglia in a sex-specific manner. *Cell* 172 500.e16–516.e16. 29275859 10.1016/j.cell.2017.11.042 PMC5786503

[B47] Vetere G. Kenney J. W. Tran L. M. Xia F. Steadman P. E. Parkinson J. (2017). Chemogenetic interrogation of a brain-wide fear memory network in mice. *Neuron* 94 363.e4–374.e4. 28426969 10.1016/j.neuron.2017.03.037

[B48] Wang T. Hu X. Liang S. Li W. Wu X. Wang L. (2015). Lactobacillus fermentum NS9 restores the antibiotic induced physiological and psychological abnormalities in rats. *Benef. Microbes* 6 707–717. 10.3920/bm2014.0177 25869281

[B49] Wheeler A. L. Teixeira C. M. Wang A. H. Xiong X. Kovacevic N. Lerch J. P. (2013). Identification of a functional connectome for long-term fear memory in mice. *PLoS Comput. Biol.* 9:e1002853. 10.1371/journal.pcbi.1002853 23300432 PMC3536620

[B50] Winocur G. Wojtowicz J. M. Sekeres M. J. Snyder J. S. Wang S. (2006). Inhibition of neurogenesis interferes with hippocampus-dependent memory function. *Hippocampus* 16 296–304. 10.1002/hipo.20163 16411241

[B51] Yano J. M. Yu K. Donaldson G. P. Shastri G. G. Ann P. Ma L. (2015). Indigenous bacteria from the gut microbiota regulate host serotonin biosynthesis. *Cell* 161 264–276. 10.1016/j.cell.2015.02.047 25860609 PMC4393509

