# Supplementary material for: Disrupted Neurogenesis in Germ-Free Mice: Effects of Age and Sex
Source: Front Cell Dev Biol. 2020 May 29;8:407. doi: 10.3389/fcell.2020.00407 (PMC7272680; doi:10.3389/fcell.2020.00407)
Supplement: Supplementary file 1 [file Image_1.PDF]

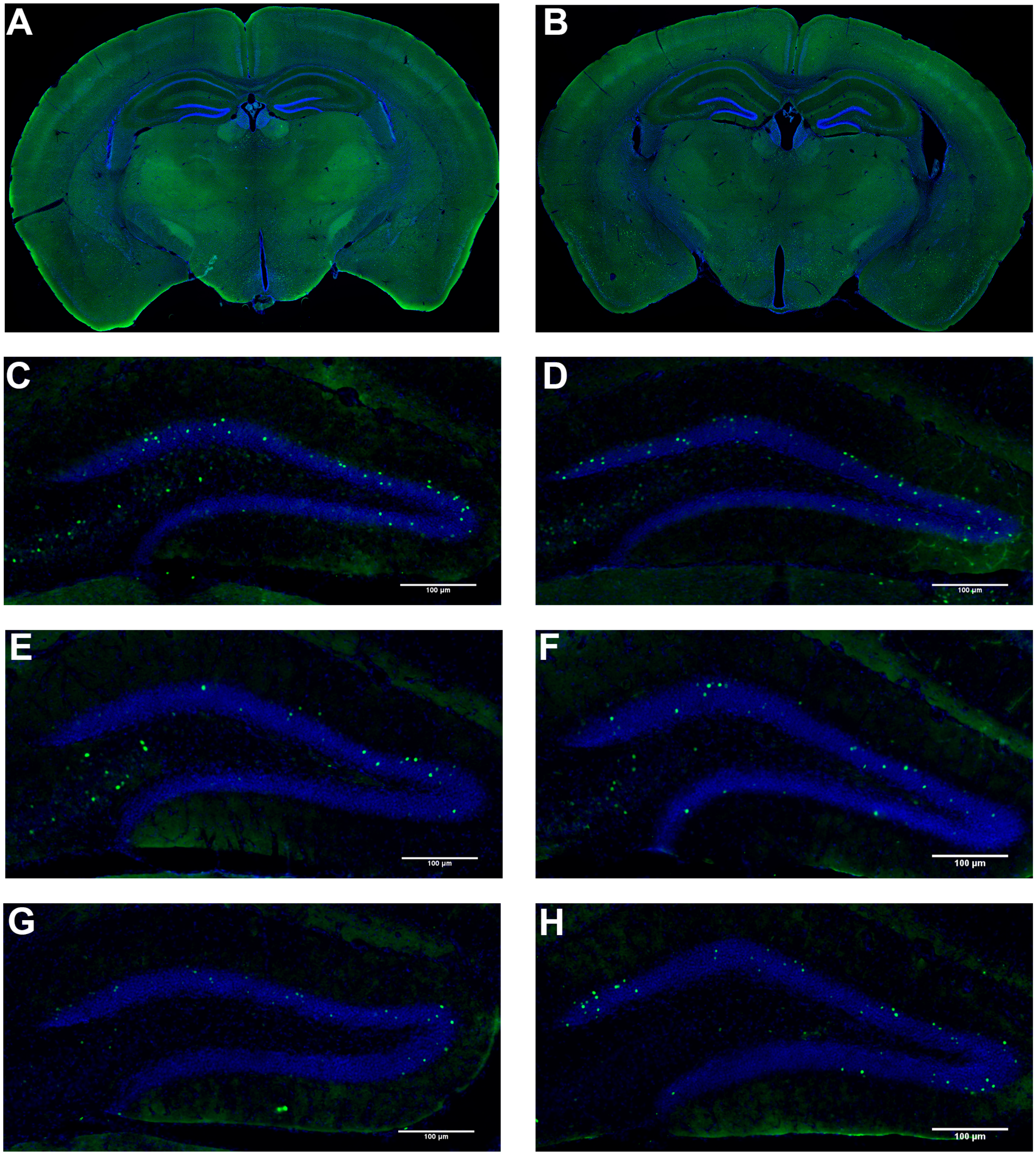

Supplementary Figure 1. A,C,E,G) Representative photomicrographs of c-fos expression in control mice, including the dentate gyri of 4-week-old (C), 8-week-old (E), and 12-week-old (G) mice. **B,D,F,H)** Representative photomicrographs of c-fos expression in germ-free mice, including the dentate gyri of 4-week-old (D), 8-week-old (F), and 12-week-old (H) mice.
