# Supplementary material for: Disrupted Neurogenesis in Germ-Free Mice: Effects of Age and Sex
Source: Front Cell Dev Biol. 2020 May 29;8:407. doi: 10.3389/fcell.2020.00407 (PMC7272680; doi:10.3389/fcell.2020.00407)
Supplement: Supplementary file 2 [file Table_1.DOCX]

Supplemental Table 1**:** List of region abbreviations used for network analysis and their full names.

| Abbreviation | Full Region Name |
| --- | --- |
| ACA | Anterior cingulate area |
| AI | Anterior insular area |
| AUD | Auditory areas |
| BLA | Basolateral amygdalar nucleus |
| CA1 | Field CA1 |
| CA2 | Field CA2 |
| CA3 | Field CA3 |
| CA4 | Field CA4 |
| COA | Cortical amygdalar area |
| CP | Caudoputamen |
| DG | Dentate gyrus |
| ECT | Ectorhinal area |
| ENT | Entorhinal area, lateral part |
| ENTm | Entorhinal area, medial part, dorsal zone |
| ENTmv | Entorhinal area, medial part, ventral zone |
| LD | Lateral dorsal nucleus of the thalamus |
| M1 | Primary motor area |
| M2 | Secondary motor area |
| MD | Mediodorsal nucleus of the thalamus |
| PAA | Piriform-amygdalar area |
| PAR | Parasubiculum |
| PERI | Perihinal area |
| PIR | Piriform area |
| POST | Postsubiculum |
| PRE | Presubiculum |
| PTL | Posterior parietal association areas |
| PVT | Paraventricular thalamic nucleus |
| RSA | Retrosplenial area, agranular part |
| RSG | Retrosplenial area, granular part |
| SS | Somatosensory areas |
| SUBd | Subiculum, dorsal part |
| SUBv | Subiculum, ventral part |
| TEA | Temporal association areas |
| VIS | Visual areas |
| VISC | Visceral areas |
